# Durable Remission with Immunotherapy in a Patient with Sarcomatoid Renal Cell Carcinoma

**DOI:** 10.15586/jkcvhl.v8i4.168

**Published:** 2021-10-26

**Authors:** Anusim Nwabundo, Gbadebo Damilola, Afolayan-Oloye Olabisi, Jaiyesimi Ishmael

**Affiliations:** Department of Hematology and Oncology, Beaumont Health, Royal Oak, MI, USA

**Keywords:** case study, immunotherapy, renal cell carcinoma, sarcomatoid differentiation

## Abstract

Sarcomatoid differentiation is a rare and aggressive histologic subtype with poor prognosis, seen in several malignancies. In sarcomatoid renal cell carcinoma (RCC), the degree of sarcomatoid differentiation and the stage at presentation determines the prognosis. Despite resection, chemotherapy and targeted therapy response is modest, with relapse usually occurring within a few months. We present a case of a gentleman with sarcomatoid RCC managed with pembrolizumab, who has had no evidence of recurrence for over 4 years since the last dose of immunotherapy. RCCs with sarcomatoid differentiation have a high presence of programmed cell death protein 1 and programmed cell death ligand 1 in T cells and tumor cells, respectively, making immunotherapy an attractive option in this setting. Clinical trials are ongoing to further define the benefit of immunotherapy in sarcomatoid RCC.

## Case Presentation

**Figure 1:**
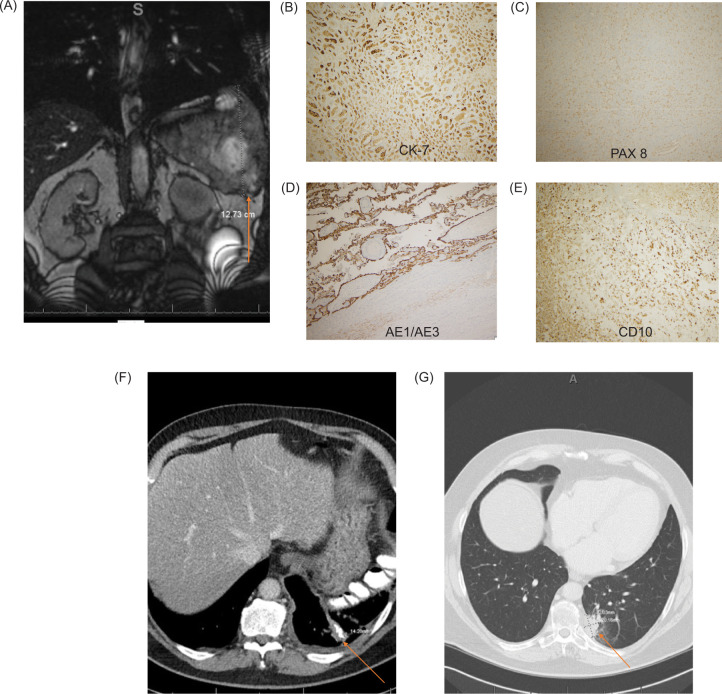
Radiologic and pathologic findings.

A 73-year-old man presented to our facility with complaints of left upper quadrant pain and twenty-pound weight loss. A computed tomographic (CT) scan of the chest, abdomen, and pelvis revealed a huge upper quadrant complex mass (Figure 1A), whereas a biopsy of the mass revealed high-grade malignant neoplasm. He underwent radical nephrectomy, splenectomy, and resection of the diaphragm. Pathology revealed a 23.5 cm mass with histologic grade 4 renal cell carcinoma (RCC) with sarcomatoid (95%) and focal papillary (1%) features that invaded into the perinephric fat, gerota fascia, spleen, and diaphragm, all with negative margins, translating to a pathological stage 4. On immunohistochemistry, the tumor stained positive for CK 7, PAX-8, and CD-10, whereas AE1/AE3 was focally positive (Figure 1B–E). Three months postnephrectomy, he complained of pleuritic chest pain. A CT scan of the chest revealed abnormal tissue in the inferior posterior left pleural space, and left-sided abdominal and peritoneal lymph nodes. Biopsy revealed sarcomatoid RCC. He received radiation therapy to that region and subsequent chemotherapy with sunitinib and gemcitabine. A repeat CT of the chest, abdomen, and pelvis 6 months after therapy initiation revealed no evidence of disease.

Unfortunately, 9 months after chemotherapy initiation, the patient noted a tender subcutaneous nodule in the left chest wall. CT scan of the chest revealed a new irregular area of pleural soft tissue at the posterior medial aspect of the left lower lobe of the lung (Figure 1G). A positron emission tomography (PET) scan revealed multiple suspicious retrocrural and left aortic lymph nodes with intense 18F-fluorodeoxyglucose (FDG) avidity, in addition to several FDG-avid soft tissue lesions in the left chest and abdominal wall. He underwent radiation to the left abdominal wall and due to his high PD-1 and PD-L1 status he was prescribed pembrolizumab, which he received for only 3 months because of grade 4 immune-mediated pneumonitis. Repeat PET scans showed interval resolution of the previous lesions in the peritoneal and retroperitoneal cavities and the posterior left costophrenic angle, indicating a favorable response to therapy. Recent PET scans after more than 4 years of immunotherapy revealed no evidence of recurrent disease.

## Discussion

Sarcomatoid RCC is a rare histologic subtype, present in 5%-8% of all RCCs (1). It is aggressive with a poor prognosis and overall survival of approximately 4 months when diagnosed at an advanced stage (2). Sarcomatoid RCC can also be seen in combination with other subtypes of RCC, where a higher percentage of sarcomatoid features confers a worse prognosis (3). Surgical resection is curative in early-stage disease. However, in advanced disease, there is typically a rapid onset of relapse despite complete surgical resection. In a retrospective review of patients with metastatic chromophobe RCC, patients with sarcomatoid features versus those without had a statistically significant shorter median time to metastatic disease relapse after nephrectomy for localized disease (2.7 months vs. 48.8 months) and treatment failure during first-line treatment (1.8 months vs. 8 months) when compared with those without sarcomatoid features (4). Chemotherapeutic agents have limited success in producing response or durable remission. In a phase 2 trial of doxorubicin and gemcitabine in 39 patients with sarcomatoid RCC, 16% of patients experienced response, whereas 26% had stable disease. The median progression-free survival (PFS) was 3.5 months, and the median overall survival (OS) was 8.8 months (5). Tyrosine kinase inhibitors, mammalian target of rapamycin (mTOR) inhibitors, and vascular endothelial growth factor inhibitors with chemotherapy have been utilized with modest improvement in survival (6,7). In a phase 2 trial of capecitabine, gemcitabine, and bevacizumab in patients with metastatic or unresectable sarcomatoid RCC, the response rate remained low, with only 20% of the participants achieving a response and only one patient with a complete response. The median PFS in this trial was 5.5 months, and the median OS was 12 months. About 91% of patients discontinued treatment because of treatment-related toxicity, commonly noted with combination therapies (7). In a retrospective study of patients with sarcomatoid RCC who received sunitinib, PFS was 5.7 months, with only 30% of patients achieving stable disease (1). In another retrospective study assessing the benefit of mTOR inhibitors in 23 patients with sarcomatoid RCC, the median PFS and OS were 3.5 months and 8.2 months, respectively (6).

RCCs with sarcomatoid differentiation have been noted to have 89% programmed cell death ligand 1 (PDL-1) positivity and combined PDL-1 and programmed cell death protein-1 positivity of 50% (8). Despite this, studies have shown conflicting responses to immunotherapy. In a retrospective review of patients with sarcomatoid RCC treated with immunotherapy versus chemotherapy, a triple improvement in the median PFS in the immunotherapy group and no difference in OS was observed (9). In a single case report, a notably durable remission of about 2 years was seen in a patient who received nivolumab (10). Our patient achieved an ongoing 4-year remission after treatment with pembrolizumab.

The phase 3 Checkmate 426 trial comparing the combined targeted agents axitinib and pembrolizumab versus sunitinib in 105 patients with sarcomatoid RCC showed impressive results in favor of combined axitinib and pembrolizumab. There was an almost doubling of the objective response rate (ORR, 58.8% vs. 31.5%) and a complete response rate (CRR) of 11.8% vs. 0%. The 12-month overall OS showed a 42% reduction in the risk for death in favor of pembrolizumab and axitinib (83.4% vs. 79.5%) (11).

In the recent phase 3 Checkmate 214 trial, which compared dual immunotherapy (nivolumab and ipilimumab) with sunitinib, in patients with advanced RCC with sarcomatoid features the median OS was not reached at a minimum follow-up of 42 months in the immunotherapy arm compared with 14.2 months in the sunitinib arm, which translated to a statistically significant 55% reduction in the risk for death with dual immunotherapy. In addition to the impressive OS benefit, the ORR and CRR were 2.6 and 6.1 times higher, respectively, than with sunitinib (60.8% vs. 23.1% and 18.9% vs. 3.1%) (12).

Considering these impressive data, an immune checkpoint-based regimen should be the initial choice in the management of patients with metastatic or advanced sarcomatoid RCC over local therapies like nephrectomy and radiation therapy.

Patients are encouraged to participate in clinical trials for further treatment developments with improved efficacy and limited toxicities. Currently, clinical trials are in progress using immunotherapy and immunotherapy in combination with targeted agents to define the benefits of immunotherapy in sarcomatoid RCC. However, patient accrual in these trials may be difficult because of the rarity of this histologic subtype. Umbrella trials are an effective means of investigating different cancers with sarcomatoid histology to assess biomarkers and treatment options (Table 1).

**Table 1:** Clinical trials including tumors with sarcomatoid histology, ongoing as of December 29, 2020

Trial	NCT Number	Title	Status	Phase	Interventions	Outcome Measures:
1	NCT04272034	Safety, Tolerability, Pharmacokinetics, and Pharmacodynamics of INCB099318 in Participants With Advanced Solid Tumors	Not yet recruiting	1	Drug: INCB099318	Safety and pharmacokinetics
2	NCT03866382	Testing the Effectiveness of Two Immunotherapy Drugs (Nivolumab and Ipilimumab) With One Anti-cancer Targeted Drug (Cabozantinib) for Rare Genitourinary Tumors	Recruiting	2	Drug: Cabozantinib S-malate Biological: Ipilimumab Biological: Nivolumab	ORR, DR, PFS, OS, Safety Clinical benefit rate
3	NCT03793166	Immunotherapy With Nivolumab and Ipilimumab Followed by Nivolumab or Nivolumab With Cabozantinib for Patients With Advanced Kidney Cancer, The PDIGREE Study	Recruiting	3	Drug: Cabozantinib Biological: Ipilimumab Biological: Nivolumab	OS, PFS, CR, ORR, Safety
4	NCT03685448	ANZUP - Non-clear Cell Post Immunotherapy CABozantinib (UNICAB)	Recruiting	2	Drug: Cabozantinib	ORR, PFS, Safety The number of patients alive at end of the study, assessed by date of death
5	NCT03177239	Phase II Sequential Treatment Trial of Single Agent Nivolumab, Then Combination Ipilimumab + Nivolumab in Metastatic or Unresectable Non-Clear Cell Renal Cell Carcinoma (ANZUP1602)	Active, not recruiting	2	Drug: Nivolumab Drug: Ipilimumab	OTRR,dTORR, PFS, IRTRR, IRDCR Safety
6	NCT03055013	Nivolumab in Treating Patients With Localized Kidney Cancer Undergoing Nephrectomy	Recruiting	3	Procedure: Conventional Surgery Biological: Nivolumab	EFS, OS,RFS, incidence of toxicity
7	NCT02496208	Cabozantinib S-malate and Nivolumab With or Without Ipilimumab in Treating Patients With Metastatic Genitourinary Tumors	Recruiting	1	Drug: Cabozantinib S-malate Biological: Ipilimumab Biological: Nivolumab	Recommended phase II dose (Phase I), Safety, RR, PFS, OS PDL-1 and MET expression

ORR, Objective response rate; dORR; Duration of objective response; DR, Duration of response; PFS, Progression-free survival; OS, Overall survival; CR, Complete response; OTRR, Objective tumor response rate; dOTRR, Duration of objective tumor response; IRTRR,Immune-related tumor response rate; IRDCR, Immune-related disease control rate.
